# Electronic Structure of Nitrogen- and Phosphorus-Doped Graphenes Grown by Chemical Vapor Deposition Method

**DOI:** 10.3390/ma13051173

**Published:** 2020-03-06

**Authors:** L. G. Bulusheva, V. E. Arkhipov, K. M. Popov, V. I. Sysoev, A. A. Makarova, A. V. Okotrub

**Affiliations:** 1 Nikolaev Institute of Inorganic Chemistry SB RAS, 3 Acad. Lavrentiev Ave., Novosibirsk 630090, Russia; slavaarhipov@ngs.ru (V.E.A.); popov@niic.nsc.ru (K.M.P.); sysoev@niic.nsc.ru (V.I.S.); spectrum@niic.nsc.ru (A.V.O.); 2 Department of Natural Sciences, Novosibirsk State University, 2 Pirogova Str., Novosibirsk 630090, Russia; 3 Physical Chemistry, Institute of Chemistry and Biochemistry, Free University of Berlin, 14195 Berlin, Germany; anna.makarova@helmholtz-berlin.de

**Keywords:** few-layer graphene, doping, nitrogen, phosphorus, CVD, electronic structure, resistivity

## Abstract

Heteroatom doping is a widely used method for the modification of the electronic and chemical properties of graphene. A low-pressure chemical vapor deposition technique (CVD) is used here to grow pure, nitrogen-doped and phosphorous-doped few-layer graphene films from methane, acetonitrile and methane-phosphine mixture, respectively. The electronic structure of the films transferred onto SiO_2_/Si wafers by wet etching of copper substrates is studied by X-ray photoelectron spectroscopy (XPS) and near-edge X-ray absorption fine structure (NEXAFS) spectroscopy using a synchrotron radiation source. Annealing in an ultra-high vacuum at ca. 773 K allows for the removal of impurities formed on the surface of films during the synthesis and transfer procedure and changes the chemical state of nitrogen in nitrogen-doped graphene. Core level XPS spectra detect a low *n*-type doping of graphene film when nitrogen or phosphorous atoms are incorporated in the lattice. The electrical sheet resistance increases in the order: graphene < P-graphene < N-graphene. This tendency is related to the density of defects evaluated from the ratio of intensities of Raman peaks, valence band XPS and NEXAFS spectroscopy data.

## 1. Introduction

Planar graphene sheets possessing a good electrical conductivity and mechanical strength as well as transparency and flexibility are very attractive for advanced electronics and energy applications [1,2]. The chemical vapor deposition (CVD) method provides a scalable synthesis of the sheets on different catalytic substrates, and copper is among the cheapest ones [3]. At elevated temperatures, precursor carbon-containing molecules decompose at the substrate and the obtained species form the graphene domains that coalescence over the synthesis yields a continuous polycrystalline graphene layer [4]. The low solubility of carbon in copper allows the production of large area graphene monolayers on the substrates [5]. Few-layer graphene is also an important material for the applications, which require specific mechanical properties and interlayer spaces [6]. It is assumed that the growth of a few layers on a copper substrate is due to the penetration of feeding gas molecules between copper and underlayer graphene [7]. It becomes possible when edges of graphene sheet are not in tight contact with copper. A high ratio of hydrogen gas in the reaction mixture could provide saturation of the edges by hydrogen atoms [8], however the exact synthesis mechanism is still unknown [9].

The use in the CVD process of molecules containing non-carbon atoms is a practical way to initiate the heteroatom doping of graphene [10,11]. The insertion of foreign elements into the graphene lattice changes the electronic properties and enhances the reactivity of graphene [12], which is rather inert chemically. Nitrogen is the most often used doping element. There are two main reasons for this: (1) the close size of the nitrogen atom to the carbon one makes the substitution easier and (2) many nitrogen-containing compounds can be used in CVD synthesis [10,13], where volatile reagents are required. The highest concentration of nitrogen achieved in the N-graphene is ca. 16.7% [14]. As for the phosphorus, it buckles significantly out of the graphene plane due to the large size of the atom [15]. This complicates the CVD synthesis of P-graphene and so far, there are only a few works on this topic [16,17,18]. The values of the content of phosphorus in P-graphene reported in the literature are below ca. 5%. 

Direct substitution of carbon by nitrogen results in the *n*-doping of graphene [19]. This form of the inserted nitrogen, so-called graphitic N, is the most preferable energetically [20]. At low synthesis temperatures, pyridinic N and pyrrolic N forms are usually realized [21,22]. The former nitrogen is a two-coordinated atom located at boundaries of vacancies or graphene domains, while the latter nitrogen is in a carbon pentagonal ring and bonded with hydrogen atom. The quantum-chemical calculations predict *p*-type graphene doping with the insertion of pyridinic N and no electron/hole doping by pyrrolic N [23]. Surprisingly, most of the literature data detect the larger conductivity of the CVD-produced N-graphene, as compared to pure graphene independently on the form of the incorporated nitrogen [24,25,26]. Phosphorus is less electronegative than carbon and therefore it should donate electron density to graphene [27]. The studies of the electrical properties of P-graphene films observed the *n*-type doping of graphene with substitutional phosphorus [16,28,29].

In current work, we report the synthesis of few-layer graphene films on copper substrates using low pressures of a gaseous mixture of CH_4_/H_2_ at 1323 K (pure graphene), CH_3_CN/H_2_ at 1123 K (N-graphene) and CH_4_/PH_3_/H_2_ at 1173 K (P-graphene). The films transferred onto the SiO_2_/Si substrates were studied by Raman, X-ray photoelectron spectroscopy (XPS), near-edge absorption fine structure (NEXAFS) spectroscopy and electrical conductivity measurements. We showed that an annealing of the films in a vacuum is a necessary step to remove the surface contaminations and it modifies the electronic state of the constituting elements. The changes in the sheet resistance were related with the alternations observed experimentally in the valence and conduction bands of pure graphene film upon the insertion of nitrogen and phosphorus.

## 2. Materials and Methods 

Graphene films were grown on electrolytic 35 µm thick copper foils (Chang Chun Group, Taipei, Taiwan) using the low-pressure variant of the CVD method. Details of the pretreatment of copper foil and the design of tubular CVD reactor equipped with a moveable oven can be found in [30]. One square centimeter foil was placed in the reactor center. The reactor was evacuated to a pressure of 5 Pa and filled with hydrogen to a pressure of 10000 Pa. To remove the surface oxide and enlarge the copper grain size, the foil was annealed at 1323 K for 0.3 h, cooled to ca. 723 K at a rate of ca. 0.5 grad min^−1^ and then naturally to ca. 423 K. Then, the reactor was evacuated, filled with a carbon-containing gas and hydrogen to pressure values of 400 Pa and 2000 Pa, respectively, and heated to a required temperature. The synthesis was conducted for 0.3 h, and after that the oven was moved away from the foil and the reactor was cooled down at a rate of ca. 2 grad min^−1^. Pure graphene was synthesized from methane at 1323 K. 40 Pa of phosphine was added to methane for the growth of P-graphene at 1123 K, and N-graphene was produced from acetonitrile at 1173 K [31]. 

The copper foil was etched in an aqueous solution of iron (III) chloride (30 wt.%) for 2 h. Cooper-free graphene film was washed twice in diluted HCl (10 wt.%) and then in deionized water to neutral pH. The floating graphene was caught by a silicon substrate with a 300-nm surface oxide layer. The sample was dried in laboratory conditions. 

The images of the films on SiO_2_/Si were taken using a BX 51TRF (Olympus Corporation, Tokyo, Japan) optical microscope and S-3400N (Hitachi Ltd., Berkshire, UK) scanning electron microscope (SEM). Raman spectra were recorded using an excitation from an Ar^+^ laser at 514 nm on a LabRAM HR Evolution (Horiba, Kyoto, Japan) spectrometer. Electrical characterization of the films was carried out on a four-point probe station MPS150-C1A (Cascade Microtech GmbH, Thiendorf, Germany) by Van der Pauw method under ambient conditions. Electrodes were deposited by silver conductive paint on each corner of graphene film, with a size of ca. 5 × 5 mm^2^.

The XPS and NEXAFS experiments were carried out using monochromatized synchrotron radiation from the Russian–German beamline (RGBL) at the Berliner Elektronenspeicherring für Synchrotronstrahlung (BESSY II, the Helmholtz-Zentrum Berlin, Germany). The angle between the incident radiation and the graphene surface was 55°. NEXAFS spectra near the C K-edge were acquired in the total electron yield (TEY) mode. The base pressure in the measurement chamber was better than 10^−9^ mbar. An excitation photon energy was 830 eV for XPS overall spectra and C 1s spectra, 830 and 500 eV for N 1s spectra, and 200 eV for P 2p spectra. The spectra were fitted using a Gaussian/Lorentzian function, except of the sp^2^-component in C 1s spectra, where a Doniach–Sunjic high-energy tail was added to that function. Valence-band XPS spectra were measured at 100 eV. Energies of XPS spectral lines were calibrated to the Au 4f_7/2_ component position with an accuracy of ca. 0.05 eV. 

## 3. Results

**Figure 1:**
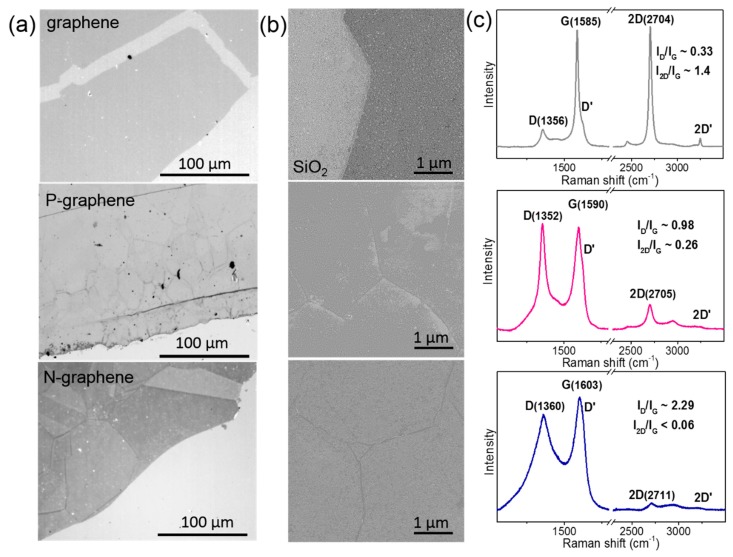
Structural characterization of graphene film (top), P-graphene film (center) and N-graphene film (bottom) located on SiO_2_/Si substrates: (**a**) Optical images; (**b**) SEM images; (**c**) Raman spectra measured at 514 nm.

The graphene monolayer located on a SiO_2_/Si substrate is visible, owing to the absorption of ca. 2.3% of white light [32]. Figure 1a compares the optical images of the transferred films. Graphene film has uniform contrast, whereas a domain structure is well distinguished in P-graphene and N-graphene. Some domains have different contrasts. This is especially visible for N-graphene and indicates the various number of graphene layers. The size of domains varies from ca. 8 to 50 µm in P-graphene and from ca. 30 to 100 µm in N-graphene. The SEM study confirms the homogeneous structure of the graphene film and the presence of domain boundaries in P-graphene and N-graphene (Figure 1b). A high magnification of the SEM instrument allows for the detection of that size of domains, which can be less than one micrometer.

It has been shown previously that the determining step for the formation of continuous graphene film is the attachment of carbon species to the graphene edges [33]. The process has a high activation energy and therefore it should occur more easily at high temperatures. Actually, the synthesis at 1323 K from methane produced a uniform film (top images in Figure 1a,b). The CVD growth of graphene includes the processes of molecule adsorption, bond breaking, carbon coalescence and other phenomena [34]. A study of the adsorption and dehydrogenation of CH_4_ on copper substrate within the density functional theory (DFT) showed that these processes rarely occur below 1073 K [35]. For the growth of P-graphene, where methane was the main feeding gas, the temperature was reduced to 1123 K to provide the insertion of phosphorus into the graphene lattice. The film formed at this low temperature consists of domains of different sizes (central image in Figure 1a). The decomposition of phosphine creates phosphorous impurities on the substrate, which can be obstacles for producing high quality graphene [34]. Compared to P-graphene, domains of N-graphene film produced at 1173 K have a larger size on average and this observation may indicate that the synthesis temperature is a more important factor for the continuous growth of graphene than the nature of the precursors. Although, the latter influences the mechanism of the graphene growth. 

Raman spectroscopy is an indispensable method for the structural characterization of graphitic materials [36]. Raman scattering detected the lowest intensity of the D peak in the spectrum of pure graphene film (Figure 1c). This peak is activated by disorder in the graphene lattice and the integrated intensity ratio of D and G peaks (I_D_/I_G_) is often used for the estimation of the density of the defects. Topological defects, edge states and stacking faults of the layers are most likely responsible for the D peak activation in pure graphene film. The intravalley phonon scattering at defects produces the D’ peak [37]. Peaks 2D and 2D’ are overtones of peaks D and D’, respectively. They are always present in the Raman spectrum, regardless of the occurrence of defects in the graphitic material. The spectra of P-graphene and N-graphene show a growth of the relative intensities of D and D’ peaks, as compared to the spectrum of pure graphene film.

Phonon scattering is dependent on electrons and any variation of electronic structure modifies the Raman peaks [37]. An upshift of the G peak in the spectra of P-graphene and N-graphene (Figure 1c) demonstrates the occurrence of doping compared to pure graphene film [38]. A larger doping level was achieved in N-graphene. A study of the electrochemically top-gated graphene showed that the shift of the 2D peak determines the type of the doping. As relative to the 2D peak for pure graphene film, the position of the 2D peak in the spectrum of P-graphene remains unchanged within the experimental error (±2 cm^−1^ at the used wavelength) and this corresponds to low electron-doping [39]. The upshift of this peak by ~7 cm^−1^ for the N-graphene could be due to hole-doping. Moreover, it was shown that the G and 2D peaks shift to the same side for hole-doping [38] and such a trend is observed in the spectrum of N-graphene. The ratio of intensities of the 2D and G peaks (I_2D/G_) is a parameter dependent on doping density [39]. This ratio is especially low for N-graphene (Figure 1c), indicating a higher doping. It was shown that the I_2D_/I_G_ value may be less than unity when the concentration of nitrogen in graphene monolayer is ~0.25–0.35% [40]. Since Raman spectra of P-graphene and N-graphene exhibited a high relative intensity of D-peak and optical images detected many domain boundaries, we suppose that both edge graphene defects and heteroatom defects are responsible for the suppression of 2D-peak intensity. 

**Figure 2:**
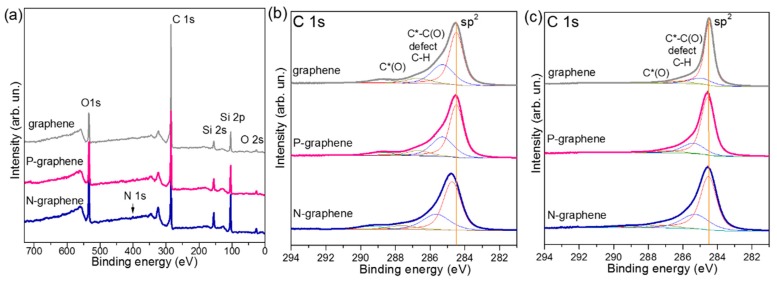
(**a**) Overall X-ray photoelectron spectroscopy (XPS) spectra of pure graphene, P-graphene, and N-graphene films annealed in spectrometer chamber at 773 K; Comparison of C 1s XPS spectra of pure graphene, P-graphene, and N-graphene films measured before (**b**) and after (**c**) annealing.

The number of layers in graphene films was evaluated from overall XPS spectra after annealing the samples in the spectrometer chamber at 773 K for 1 h. The spectra exhibited a dominant C 1s line and the core lines of oxygen and silicon from the oxidized silicon surface (Figure 2a). The higher intensities of Si 2p and Si 2s lines correspond to the thinner graphene film. Under the used synthesis conditions, we produced a thick pure graphene film and a thin N-graphene film. By taking into account a ratio of intensities of the C 1s line to the Si 2p line and atomic cross-section factors, the thickness of the films was evaluated from the attenuation law for photons in matter. The obtained values correspond to five layers in pure graphene film, four layers in P-graphene film, and three layers in N-graphene film, on average. This confirms that the I_2D_/I_G_ value from Raman scattering cannot be used to determine the thickness of heteroatom-doped graphene. 

Figure 2b presents the C 1s XPS spectra for the films on SiO_2_/Si substrates before annealing in a vacuum. In addition to an intense component corresponding to sp^2^-hybridized carbon areas, the spectra have a set of components at higher binding energies. The component at ca. 284.9 eV in the spectrum of pure graphene film is attributed to disordered carbon, which is intermediate between sp^2^ and sp^3^ states [41]. These can be topological defects constituting the boundaries of graphene domains [42], C-H bonds, and atoms located near the carbon bonded with oxygen groups (C*-C(O)) [43]. The high-energy components correspond to C–OH (286–286.7 eV), C=O (287.6–288.8 eV) and COOH (ca. 289.4 eV) groups. The oxygen species contaminating the graphene surface were developed during the procedure of film transferring from copper to SiO_2_/Si and/or contact with laboratory air. 

The disordered carbon component is upshifted by ca. 0.4 eV in the spectrum of P-graphene and ca. 0.7 eV in the spectrum of N-graphene (Figure 2b). The bonding of carbon atoms with phosphorus and nitrogen could cause this shift. Actually, the binding energies of carbon in C–P bonds are around 285.7 eV [44] and those in sp^2^ C=N and sp^3^ C–N bonds are around 285.8 and 287.1 eV, respectively [45]. These values are within the intervals occupied by the disordered components in the C 1s spectra of P-graphene and N-graphene. We also notice a marked upshift of the sp^2^ component in the spectrum of N-graphene. The shift of the C 1s core line is attributed to *p*- or *n*-type doping when the binding energy decreases or increases, respectively. However, the contact of carbon atoms with highly electronegative oxygen can be another factor causing a positive charging of the graphene network. The same shift of the sp^2^ and disordered components observed in the spectrum of N-graphene, relative to the positions of corresponding components for pure graphene, may be due to the influence of oxygen. 

Annealing in an ultra-high vacuum at 773 K strongly reduces the amounts of oxygen groups and disordered carbon in the graphene films and changes the position of sp^2^ components (Figure 2c). A higher relative intensity of high-energy components in the spectra of P-graphene and N-graphene indicates a larger disorder in these films as compared to the film of pure graphene. The width of the sp^2^ component in the spectra of the annealed samples increases from 0.61 eV for graphene to 0.91 for P-graphene and 1.12 eV for N-graphene. The broadening of this component is related to a distribution of different orientations of dangling bonds in the graphitic structure [41]. The upshift of the sp^2^ component in the spectra of annealed N-graphene and P graphene is less than 0.1 eV, respective to the position for pure graphene (Figure 2c). This value is too small for us to unambiguously conclude about the *n*-type doping. 

**Figure 3:**
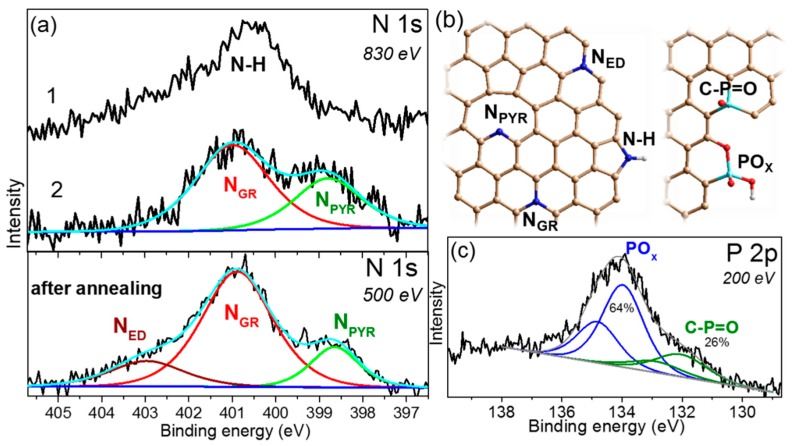
(**a**) N 1s XPS spectra of N-graphene film measured at an excitation energy of 830 eV before (1) and after (2) annealing in spectrometer chamber at 773 K and at 500 eV after annealing; (**b**) Models of N-graphene and P-graphene with heteroatoms (nitrogen shown by blue and phosphorus shown by light blue) identified by XPS; (**c**) P 2p XPS spectrum of P-graphene film measured at an excitation energy of 200 eV after annealing.

The N 1s XPS spectra of N-graphene showed that the annealing significantly affects the chemical state of nitrogen (Figure 3a). A peak at ca. 400.6 eV with a high asymmetry at both sides dominated in the spectrum of the non-annealed N-graphene/SiO_2_/Si sample. This binding energy is assigned to pyrrolic N [45]. Similar asymmetric-shape spectra have been presented previously in the papers devoted to the nitrogen-doped graphenes [24,25,46,47,48]. After the sample annealing at 773 K, the N 1s spectrum exhibited two well-resolved peaks at 398.7 and 401.0 eV, corresponding to pyridinic N and graphitic N, respectively. The total concentration of nitrogen in the annealed N-graphene film was evaluated from the overall XPS spectrum (Figure 2a) to be about 0.7%. 

The studies of nitrogen-containing graphitic materials have detected the lowest thermal stability of the pyrrolic N [49,50] and here we show that the used temperature is sufficient for the de-hydrogenation of this nitrogen. A rise of the low-energy component and the high-energy component in the spectrum of the annealed N-graphene supposes the transformation of the pyrrolic N in the pyridinic N and graphitic N, respectively. At the excitation energy of 830 eV, the mean free path of the N 1s photoelectrons allows probing about five graphene layers [51]. Thus, the spectra measured at this energy provide information about nitrogen distributed in all layers of the N-graphene film. 

With the purpose of examining the electronic state of nitrogen in two surface layers, we measured the N 1s spectrum at an excitation energy of 500 eV (Figure 3a). As compared to the spectrum recorded at 830 eV, this spectrum showed a reduced intensity of the peak at 398.7 eV and emergence of a high-energy peak at ca. 403 eV. The latter peak is often attributed to oxidized nitrogen species [52]. However, such an assignment is unacceptable in this case because the N-graphene sample has been annealed in an ultra-high vacuum and after that its surface was not expose to air. Other possible configurations of nitrogen, which can possess this binding energy, are the clustered graphic N atoms [45] and graphitic N located at the sheet edge [53]. These atoms should be more stable energetically than the pyridinic N. Nitrogen forms, which can be realized in the studied N-graphene films, are schematically shown in Figure 3b. 

Figure 3c presents the P 2p XPS spectrum measured for the annealed P-graphene. The estimated concentration of phosphorus is below 0.1%. A low-energy spin-orbit doublet with the energy of P 2p_3/2_ component of ca. 132.0 eV is assigned to the C–P=O bonds [54,55]. An intense doublet with the P 2p_3/2_ component located at ca. 134.0 eV corresponds to the higher oxidation state of phosphorus. These oxidized phosphorus species are likely located at the edge of graphene domains as the model in Figure 3b proposes.

**Figure 4:**
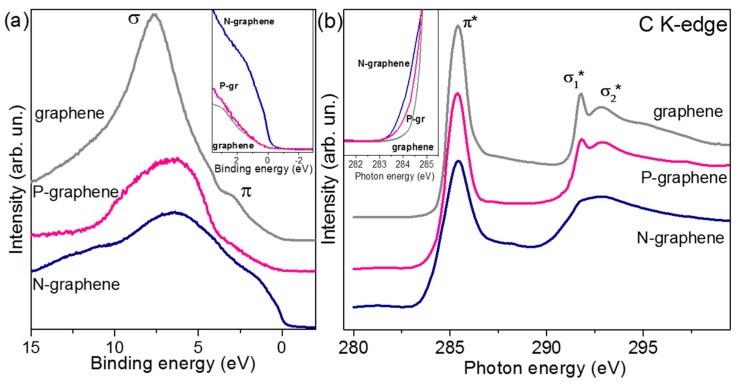
(**a**) Valence band XPS and (**b**) near-edge X-ray absorption fine structure (NEXAFS) C K-edge spectra of the annealed graphene, N-graphene, and P-graphene films. Inserts show the spectra near the Fermi level.

The valence band XPS spectra of the annealed films revealed an influence of the underlayer SiO_2_/Si substrate on the spectral shape (Figure 4a). The spectrum of pure graphene film is similar to that measured for sp^2^-hybridized carbon at the used excitation energy [56]. The dominant peak in the spectrum corresponds to occupied σ-states, and the peak at ca. 3.0 eV is formed by π-states of graphene. The spectral shape characteristic for the sp^2^ carbon is significantly distorted in the case of N-graphene film. This film is the thinnest of the studied samples that result in greater contribution from the SiO_2_ to the spectrum. A study of SiO_2_/Si samples by valence band XPS spectroscopy has detected the appearance of spectral intensity after 4 eV [57]. Therefore, we can compare effect of the heteroatom doping on the valence states before this value. The insert in Figure 4a compares the fragments of the spectra, normalized to the maximal intensity. Between 0 eV and ca. 2.5 eV, pure graphene and P-graphene have a similar density of occupied states. The nitrogen doping cannot cause a large increase of the density observed in the valence band XPS spectrum of N-graphene. It has been shown previously that the electrons of carbon atoms at the boundaries of graphene-like structures form localized states near the Fermi level [58].

Unoccupied states of carbon in graphene films were examined using NEXAFS C K-edge spectra (Figure 4b). Electron transitions from 1s carbon levels to partially occupied and empty π- and σ-states produce the π*-resonance at 285.4 eV and σ*-resonances at 291.7 and 292.8 eV [59]. Splitting of the σ*-resonance indicates a high crystallinity of graphene layers [60]. The smoothing of the splitting observed in the spectrum of N-graphene confirms that this film has the highest amount of imperfections in the structure. The absence of peaks between the π*- and σ*-resonances indicates a negligible amount of heteroatoms (including oxygen) in the annealed graphene films. A weak peak at ca. 288.2 eV can be attributed to C–N bonding [61,62]. The insert in Figure 4b compares the low-energy region of the C K-edge spectra, normalized to the π*-resonance intensity. The density of the unoccupied states gradually increases from pure graphene to P-graphene and then to N-graphene. This shoulder can be attributed to vacancy and edge defects [63]. 

**Figure 5:**
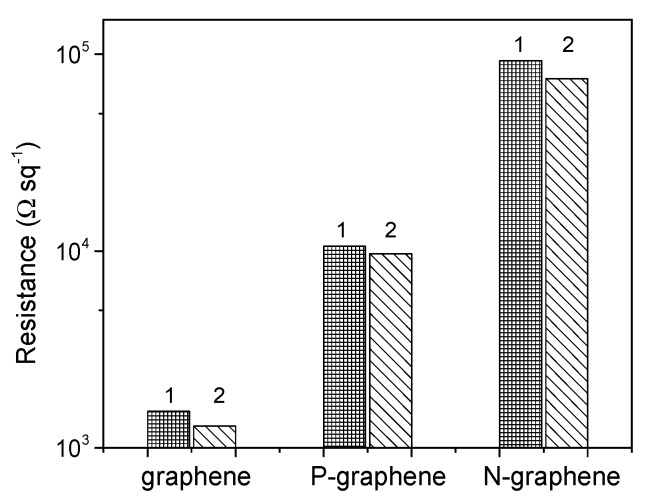
Sheet resistance of pure graphene, P-graphene, and N-graphene measured at ambient conditions before (1) and after (2), the film annealing in ultra-high vacuum at 773 K.

A study of electrical properties of the SiO_2_-supported graphene films detected a decrease in sheet resistance after annealing (Figure 5). The literature data show that graphene easily interacts with air and the adsorbed oxygen molecules cause *p*-type doping particularly [64]. Our annealed samples were stored for a few days in ambient conditions before the measurements of electrical properties. During this time, they interacted with environmental air molecules, which could change sheet resistance to the initial value. However, that did not happen. Hence, the imperfections produced during the CVD synthesis and the contaminations from the transfer procedure also have an impact on charge transport in graphene films. The sheet resistance increases as graphene < P-graphene < N-graphene. This behavior may correspond to the *n*-type doping of P-graphene and N-graphene compared to *p*-doped graphene. 

## 4. Discussion

The studied graphene samples were synthesized using the low-pressure CVD method with a change of the precursor, providing various atoms for the film growth and the synthesis temperature to facilitate the insertion of heteroatoms (phosphorus or nitrogen) in the graphene lattice. These variable parameters are listed in Table 1, together with structural and electrical characteristics for the films studied after their wet transfer to SiO_2_/Si substrates. The used synthesis conditions, particularly, ratio of the pressures of CH_4_ and H_2_ of 400 to 2000 Pa, cause the growth of few-layer graphene [30]. It has been shown theoretically that at a high hydrogen pressure, the hydrogen atoms terminate the edges of graphene and this produces channels between graphene and copper for the diffusion of carbon species to the substrate for the growth of the next graphene layer [8]. Our results support this mechanism. The XPS spectra of raw graphene films on SiO_2_/Si substrates detected C–H (Figure 2b) and N–H states (Figure 3a). Annealing of the films in an ultra-high vacuum at 773 K substantially reduces the amount of these bonding states. The termination of edge carbon atoms with hydrogen at a high content of H_2_ in the reaction mixture is probably a common phenomenon [65]. 

Compared to homogeneous graphene film, the P-graphene and N-graphene films have shown a domain structure (Figure 1a,b). The size of graphene domains is substantially determined by the structure of the copper substrate [3,4]. Since the pretreatment of copper foil was identical in all experiments, continuous growth of the graphene film is related to the higher temperature, facilitating the attachment of the carbon species to the graphene edges. Interestingly, the number of layers in N-graphene may vary within the same domain. This could be due to a difference in the diffusion of carbon and nitrogen atoms over copper [21]. 

Raman scattering detected a huge I_D_/I_G_ value for N-graphene compared to P-graphene (Table 1). Defects in these graphene films are boundaries of domains and heteroatoms. The analysis of XPS spectra determined that there was ca. 0.7% of nitrogen in N-graphene and less than 0.1% of phosphorus in P-graphene. The tiny shifts of the Raman peaks for P-graphene could correspond to a very low *n*-type doping, however, the value of the shifts lies within the experimental error. The spectrum of N-graphene showed the upshifts of the peaks. The same behavior was observed when a negative gate voltage applied to graphene induced its hole-doping [38]. However, an increase of the binding energy of C 1s peak in the XPS spectrum of N-graphene (Figure 2b) is a sign of *n*-type doping. Another reason for the stiffening of the Raman peaks is the distortion of carbon bonds due to insertion of nitrogen atoms in the graphene lattice [66]. Pyrrolic N introduces the greatest distortion and this form prevails in the synthesis product as the N 1s spectrum shows (Figure 3a).

Annealing of the samples in ultra-high vacuum at 773 K for 1 h modified the electronic state of carbon and nitrogen. The treatment allowed the removal of most of the oxygen groups and reducing disorder in the graphene films. Since after that the C 1s spectrum of N-graphene moved closer to the position of the pure graphene spectrum (Figure 2c), we conclude that the upshifts of the spectral peaks observed in the raw film are due to a polarization effect from oxygen groups. As the result of annealing, pyrrolic N transformed to pyridinic N and graphitic N. Graphitic N forms faster in the upper layers of the film. This result agrees with the observation of preferable formation of the graphitic N under annealing of few-layer graphene with the nitrogen species implanted by nitrogen ions irradiation [67]. Annealing did not clear the phosphorus atoms from oxygen, due to strong interactions between the atoms. 

The XPS measurements observed an upshift of the top of the valence band for N-graphene only (Figure 3a). This was accompanied by a huge increase in the density of the occupied states. The quantum-chemical calculations have predicted the appearance of a strong peak before the Fermi level and *p*-doping for N-graphene containing 1% of pyridinic N [68]. This behavior was related to the edge states of the vacancies required for pyridinic N placement. Our N-graphene after annealing contained ca. 0.3% of pyridinic N and ca. 0.4% of graphitic N. According to theory, this could cause an increase of both occupied and empty states around the Fermi level, whereas the Dirac point will slightly upshift relative to that for graphene [68]. The former effect reveals itself in XPS valence band and NEXAFS spectra as an enhanced intensity around the Fermi level (Figure 4); the latter one is observed as an upshift of the C 1s peak for N-graphene (Figure 2c).

The measurements of the electrical properties of the films detected a larger sheet resistance of graphene films with heteroatoms (Figure 5). The *n*-type doping could explain a decrease in the conductivity of P-graphene and N-graphene relative to the *p*-doped pure graphene. The analysis of the C 1s spectra suggests a higher level of doping for P-graphene, whereas the sheet resistance is substantially larger for N-graphene (Table 1). Thus, we conclude that the low conductivity comes mainly from the scattering of charge carriers by heteroatoms and topological defects on the domain boundaries [69]. Domain boundaries may give rise to the carrier scattering and serve as the adsorption sites for gas molecules and contaminations. The used spectroscopic methods observed the higher impact of defects on the electronic structure of N-graphene as compared to P-graphene. Optical microscopy, however, demonstrated a smaller size of domains on average in the latter film (Figure 1a). Therefore, we conclude that the domain boundaries in N-graphene and P-graphene have different structures. The decomposition of acetonitrile, the precursor of N-graphene, and methane mixed with phosphine, used for the growth of P-graphene, yield different species (C, C_2_, CN, N, P, etc.), which require different energies for the activation and attachment to the edges of a growing domain. As the result, the edges of the adjacent domains may connect tightly or weakly, forming a compact or sparse boundary. 

The insertion of nitrogen and phosphorous in graphene can be achieved during the synthesis or post-synthesis treatment. Chemical forms of the embedded heteroatoms and electronic structure of the obtained material depend on the synthesis method, as well as the used parameters. In the post-synthesis treatment, the nature of the initial graphene material is also important. Particularly, the treatment of few-layer graphene may introduce heteroatoms only in the surface layers. The synthesis, characterization and properties of heteroatom-doped graphene nanomaterials are described in the recent reviews [10,11,12,70]. The methods providing the bonding of carbon atoms with heteroatoms during the graphene growth are CVD and arc discharge [11,71]. There, the concentration and forms of the nitrogen atoms are strongly dependent on the nitrogen-containing precursor [72], the synthesis temperature [73], the inert gas [74], etc. The phosphorus atom is difficultly inserted into the growing graphene due to the large atomic size. Because the C–C bond is thermodynamically more preferable than C–N and C–P bonds, the increase of the synthesis temperature reduces the amount of the embedded heteroatoms [70]. Generally, the substitution of a carbon atom by nitrogen or phosphorous results in the *n*-type doping of graphene. Pyridinic N induces *p*-doping, while in the hydrogenated form it can behave as an *n*-type dopant [73]. The effect of pyrrolic N is still unclear. Graphene edges are necessary for the accommodation of these nitrogen forms and the edge states between domains and at the vacancy boundaries, which affect the electronic structure of graphene. Since different forms can be realized during the synthesis and some of them require defects in the graphene lattice, it is not easy to determine a contribution of the certain form of heteroatom on the electronic structure of graphene. In this study, we were able to correlate the data of Raman scattering, NEXAFS, and XPS with measurements of the sheet resistance of graphene, P-graphene, and N-graphene, and showed an important contribution of the edges’ states to conductive properties of CVD-grown few-layer films. We found that a temperature of 773 K is sufficient for the complete conversion of pyrrolic N to graphitic N and pyridinic N. It has been shown theoretically that the co-existence of graphitic N and pyridinic N causes a little (if any) *n*-doping of graphene [68]. Our experiments confirmed this theoretical prediction. 

## 5. Conclusions

We have compared three graphene films, synthesized by low-pressure CVD method on copper foils, pretreated in the same way. Methane and acetonitrile provided the necessary species for the growth of graphene and N-graphene, respectively, and P-graphene was produced using a mixture of methane and phosphine, taken in the ratio 10:1. The synthesis temperature was 1323 K for pure graphene, 1123 K for P-graphene and 1173 K for N-graphene. The lowering of the temperature and its addition to the reaction mixture heteroatoms resulted in the formation of different size domains in P-graphene and N-graphene. The films were successfully transferred to SiO_2_/Si substrates to study the structure by Raman spectroscopy, the electronic state by XPS and NEXAFS spectroscopy, and the electrical properties in the air at room temperature. Raman scattering revealed an increase of disorder in the graphene films with heteroatoms. The largest ratio of I_D_/I_G_ and the smallest ratio I_2D_/I_G_ were observed in the N-graphene spectrum. An upshift of the Raman peaks in this spectrum was related to the strong distortion of carbon bonds with the incorporation of nitrogen atoms and especially of pyrrolic N. The N 1s spectrum showed the predominance of this form in raw N-graphene. Annealing of the graphene samples in an ultra-high vacuum at 773 K for 1 h substantially reduced the amount of oxygen groups, developed during the wet transfer procedure and storage of samples in air environment, and defects in the graphene lattice. A comparison of the C 1s spectra of the annealed samples observed an upshift of the peaks for N-graphene and even more for P-graphene. This shift can be attributed to *n*-type doping of the graphene samples and might explain the increase of the sheet resistance, as related to pure, *p*-doped graphene. However, the change in the sheet resistance does not correlate with the doping level, estimated from the XPS data. Based on this fact, we concluded that scattering centers, created by heteroatoms and boundaries of domains, contribute to the sheet resistance. The poor connection of boundaries in N-graphene could be a reason of its lowest conductivity. NEXAFS and XPS spectra of this film detected many localized states around the Fermi level, which most likely correspond to the edges of domains and vacancies. These states can serve as adsorption sites in the sensors and scatters of phonons in devices where the control of thermal conductivity is important.

## Figures and Tables

**Table 1:** Temperature (T_S_) and precursor used for the sample synthesis, ratio of I_D_/I_G_ from Raman scattering, number of layers (N_L_), content of heteroatoms, and binding energy (BE) of C 1s peak determined from XPS data, and sheet resistance of the annealed samples.

Sample	T_S_ (K)	Precursor	I_D_/I_G_	N_L_	Heteroatom	BE (eV)	Resistance (kΩ/□)
Graphene	1323	CH_4_	0.33	~5	-	284.47	1.3
P-graphene	1123	10CH_4_/PH_3_	0.98	~4	<0.1% P	284.56	9.7
N-graphene	1173	CH_3_CN	2.29	~3	~0.7% N	284.51	75.2

## References

[1] Zhong Y., Zhen Z., Zhu H. (2017). Graphene: Fundamental research and potential applications. FlatChem.

[2] Brownson D.A.C., Banks C.E. (2012). The electrochemistry of CVD graphene: Progress and prospects. Phys. Chem. Chem. Phys..

[3] Kraus J., Böcklein S., Reichelt R., Günther S., Santos B., Menteş T.O., Locatelli A. (2013). Towards the perfect graphene membrane?—Improvement and limits during formation of high quality graphene grown on Cu-foils. Carbon N. Y..

[4] Liu H., Liu Y. (2017). Controlled Chemical Synthesis in CVD Graphene. Phys. Sci. Rev..

[5] Chen X., Zhang L., Chen S. (2015). Large area CVD growth of graphene. Synth. Met..

[6] Xue R., Abidi I.H., Luo Z. (2017). Domain size, layer number and morphology control for graphene grown by chemical vapor deposition. Funct. Mater. Lett..

[7] Jin Y., Hu B., Wei Z., Luo Z., Wei D., Xi Y., Zhang Y., Liu Y. (2014). Roles of H_2_ in annealing and growth times of graphene CVD synthesis over copper foil. J. Mater. Chem. A.

[8] Zhang X., Wang L., Xin J., Yakobson B.I., Ding F. (2014). Role of hydrogen in graphene chemical vapor deposition growth on a copper surface. J. Am. Chem. Soc..

[9] Seah C.M., Chai S.P., Mohamed A.R. (2014). Mechanisms of graphene growth by chemical vapour deposition on transition metals. Carbon N. Y..

[10] Plutnar J., Pumera M., Sofer Z. (2018). The chemistry of CVD graphene. J. Mater. Chem. C.

[11] Xu H., Ma L., Jin Z. (2018). Nitrogen-doped graphene: Synthesis, characterizations and energy applications. J. Energy Chem..

[12] Chen N., Huang X., Qu L. (2015). Heteroatom substituted and decorated graphene: Preparation and applications. Phys. Chem. Chem. Phys..

[13] Granzier-Nakajima T., Fujisawa K., Anil V., Terrones M., Yeh Y.T. (2019). Controlling nitrogen doping in graphene with atomic precision: Synthesis and characterization. Nanomaterials.

[14] Xue Y., Wu B., Jiang L., Guo Y., Huang L., Chen J., Tan J., Geng D., Luo B., Hu W. (2012). Low temperature growth of highly nitrogen-doped single crystal graphene arrays by chemical vapor deposition. J. Am. Chem. Soc..

[15] Susi T., Hardcastle T.P., Hofsäss H., Mittelberger A., Pennycook T.J., Mangler C., Drummond-Brydson R., Scott A.J., Meyer J.C., Kotakoski J. (2017). Single-atom spectroscopy of phosphorus dopants implanted into graphene. 2D Mater..

[16] Some S., Kim J., Lee K., Kulkarni A., Yoon Y., Lee S., Kim T., Lee H. (2012). Highly air-stable phosphorus-doped n-type graphene field-effect transistors. Adv. Mater..

[17] Larrude D.G., Garcia-Basabe Y., Freire F.L., Rocco M.L.M. (2015). Electronic structure and ultrafast charge transfer dynamics of phosphorous doped graphene layers on a copper substrate: A combined spectroscopic study. RSC Adv..

[18] Ovezmyradov M., Magedov I.V., Frolova L.V., Chandler G., Garcia J., Bethke D., Shaner E.A., Kalugin N.G. (2015). Chemical vapor deposition of phosphorous and boron-doped graphene using phenyl-containing molecules. J. Nanosci. Nanotechnol..

[19] Schiros T., Nordlund D., Pálová L., Prezzi D., Zhao L., Kim K.S., Wurstbauer U., Gutiérrez C., Delongchamp D., Jaye C. (2012). Connecting dopant bond type with electronic structure in n-doped graphene. Nano Lett..

[20] Sedelnikova O.V., Bulusheva L.G., Okotrub A.V. (2015). Graphitic and pyridinic nitrogen in carbon nanotubes: Energetic and polarization aspects. J. Nanophotonics.

[21] Gaillard P., Schoenhalz A.L., Moskovkin P., Lucas S., Henrard L. (2016). Growth of nitrogen-doped graphene on copper: Multiscale simulations. Surf. Sci..

[22] Sui Y., Zhu B., Zhang H., Shu H., Chen Z., Zhang Y., Zhang Y., Wang B., Tang C., Xie X. (2015). Temperature-dependent nitrogen configuration of N-doped graphene by chemical vapor deposition. Carbon N. Y..

[23] Wang B., Tsetseris L., Pantelides S.T. (2013). Introduction of nitrogen with controllable configuration into graphene via vacancies and edges. J. Mater. Chem. A.

[24] Gao H., Song L., Guo W., Huang L., Yang D., Wang F., Zuo Y., Fan X., Liu Z., Gao W. (2012). A simple method to synthesize continuous large area nitrogen-doped graphene. Carbon N. Y..

[25] Lv R., Li Q., Botello-Méndez A.R., Hayashi T., Wang B., Berkdemir A., Hao Q., Eléas A.L., Cruz-Silva R., Gutiérrez H.R. (2012). Nitrogen-doped graphene: Beyond single substitution and enhanced molecular sensing. Sci. Rep..

[26] Cui T., Lv R., Huang Z.H., Zhu H., Zhang J., Li Z., Jia Y., Kang F., Wang K., Wu D. (2011). Synthesis of nitrogen-doped carbon thin films and their applications in solar cells. Carbon N. Y..

[27] Garcia A.G., Baltazar S.E., Castro A.H.R., Robles J.F.P., Rubio A. (2008). Influence of S and P doping in a graphene sheet. J. Comput. Theor. Nanosci..

[28] He S.M., Huang C.C., Liou J.W., Woon W.Y., Su C.Y. (2019). Spectroscopic and electrical characterizations of low-damage phosphorous-doped graphene via ion implantation. ACS Appl. Mater. Interfaces.

[29] Shin D.W., Kim T.S., Yoo J.B. (2016). Phosphorus doped graphene by inductively coupled plasma and triphenylphosphine treatments. Mater. Res. Bull..

[30] Arkhipov V.E., Gusel′nikov A.V., Popov K.M., Gevko P.N., Fedoseeva Y.V., Smirnov D.A., Bulusheva L.G., Okotrub A.V. (2018). Optimization of parameters of graphene synthesis on copper foil at low methan pressure. J. Struct. Chem..

[31] Bulusheva L.G., Kanygin M.A., Arkhipov V.E., Popov K.M., Fedoseeva Y.V., Smirnov D.A., Okotrub A.V. (2017). In situ X-ray photoelectron spectroscopy study of lithium nnteraction with graphene and nitrogen-doped graphene films produced by chemical vapor deposition. J. Phys. Chem. C.

[32] Bulusheva L.G., Sedelnikova O.V., Okotrub A.V. (2016). Many-body effects in optical response of graphene-based structures. Int. J. Quantum Chem..

[33] Kim H., Mattevi C., Calvo M.R., Oberg J.C., Artiglia L., Agnoli S., Hirjibehedin C.F., Chhowalla M., Saiz E. (2012). Activation energy paths for graphene nucleation and growth on Cu. ACS Nano.

[34] Naghdi S., Rhee K.Y., Park S.Y. (2018). A catalytic, catalyst-free, and roll-to-roll production of graphene via chemical vapor deposition: Low temperature growth. Carbon.

[35] Choi J.-H., Li Z., Cui P., Fan X., Zhang H., Zeng C., Zhang Z. (2013). Drastic reduction in the growth temperature of graphene on copper via enhanced London dispersion force. Sci. Rep..

[36] Pimenta M.A., Dresselhaus G., Dresselhaus M.S., Cançado L.G., Jorio A., Saito R. (2007). Studying disorder in graphite-based systems by Raman spectroscopy. Phys. Chem. Chem. Phys..

[37] Ferrari A.C., Basko D.M. (2013). Raman spectroscopy as a versatile tool for studing the properties of graphene. Nat. Nanotech..

[38] Fates R., Bouridah H., Raskin J.-P. (2019). Probing carrier concentration in gated single, bi- and tri-layer CVD graphene using Raman spectroscopy. Carbon.

[39] Das A., Pisana S., Chakraborty B., Piscanec S., Saha S.K., Waghmare U.V., Novoselov K.S., Krishnamurthy H.R., Geim A.K., Ferrari A.C. (2006). Monitoring dopants by Raman scattering in an electrochemically top-gated graphene transistor. Nat. Nanotech..

[40] Zhao L., He R., Zabet-Khosousi A., Kim K.S., Schiros T., Roth M., Kim P., Flynn G.W., Pinczuk A., Pasupathy A.N. (2015). Dopant segregation in polycrystalline monolayer graphene. Nano Lett..

[41] Blume R., Rosenthal D., Tessonnier J.-P., Li H., Knop-Gericke A., Schlögl R. (2015). Characterizing graphitic carbon with X-ray photoelectron spectroscopy: A step-by-step approach. ChemCatChem.

[42] Bulusheva L.G., Okotrub A.V., Asanov I.P., Fonseca A., Nagy J.B. (2001). Comparative study on the electronic structure of arc-discharge and catalytic carbon nanotubes. J. Phys. Chem. B.

[43] Bulusheva L.G., Stolyarova S.G., Chuvilin A.L., Shubin Y.V., Asanov I.P., Sorokin A.M., Mel’Gunov M.S., Zhang S., Dong Y., Chen X. (2018). Creation of nanosized holes in graphene planes for improvement of rate capability of lithium-ion batteries. Nanotechnology.

[44] Li X., Qiu Y., Hu P.A. (2016). Three dimensional P-doped graphene synthesized by eco-fiendly chamical vapor deposition for oxygen reduction reactions. J. Nanosci. Nanotech..

[45] Susi T., Pichler T., Ayala P. (2015). X-ray photoelectron spectroscopy of graphitic carbon nanomaterials doped with heteroatoms. Beilstein J. Nanotechnol..

[46] Li J., Ren Z., Zhou Y., Wu X., Xu X., Qi M., Li W., Bai J., Wang L. (2013). Scalable synthesis of pyrrolic N-doped graphene by atmospheric pressure chemical vapor deposition and its terahertz response. Carbon N. Y..

[47] Wang Z., Li P., Chen Y., Liu J., Tian H., Zhou J., Zhang W., Li Y. (2014). Synthesis of nitrogen-doped graphene by chemical vapour deposition using melamine as the sole solid source of carbon and nitrogen. J. Mater. Chem. C.

[48] Capasso A., Dikonimos T., Sarto F., Tamburrano A., De Bellis G., Sarto M.S., Faggio G., Malara A., Messina G., Lisi N. (2015). Nitrogen-doped graphene films from chemical vapor deposition of pyridine: Influence of process parameters on the electrical and optical properties. Beilstein J. Nanotechnol..

[49] Scardamaglia M., Struzzi C., Aparicio Rebollo F.J., De Marco P., Mudimela P.R., Colomer J.F., Amati M., Gregoratti L., Petaccia L., Snyders R. (2015). Tuning electronic properties of carbon nanotubes by nitrogen grafting: Chemistry and chemical stability. Carbon N. Y..

[50] Liu H., Zhang Y., Li R., Sun X., Abou-Rachid H. (2012). Thermal and chemical durability of nitrogen-doped carbon nanotubes. J. Nanoparticle Res..

[51] Bulusheva L.G., Okotrub A.V., Fedoseeva Y.V., Kurenya A.G., Asanov I.P., Vilkov O.Y., Koós A.A., Grobert N. (2015). Controlling pyridinic, pyrrolic, graphitic, and molecular nitrogen in multi-wall carbon nanotubes using precursors with different N/C ratios in aerosol assisted chemical vapor deposition. Phys. Chem. Chem. Phys..

[52] Okotrub A.V., Fedorovskaya E.O., Senkovskiy B.V., Bulusheva L.G. (2015). Nitrogen species in few-layer graphene produced by thermal exfoliation of fluorinated graphite intercalation compounds. Phys. Status Solidi Basic Res..

[53] Scardamaglia M., Aleman B., Amati M., Ewels C., Pochet P., Reckinger N., Colomer J.F., Skaltsas T., Tagmatarchis N., Snyders R. (2014). Nitrogen implantation of suspended graphene flakes: Annealing effects and selectivity of sp^2^ nitrogen species. Carbon N. Y..

[54] Okotrub A.V., Kanygin M.A., Koroteev V.O., Stolyarova S.G., Gorodetskiy D.V., Fedoseeva Y.V., Asanov I.P., Bulusheva L.G., Vyalikh A. (2019). Phosphorus incorporation into graphitic material via hot pressing of graphite oxide and triphenylphosphine. Synth. Met..

[55] Hasegawa G., Deguchi T., Kanamori K., Kobayashi Y., Kageyama H., Abe T., Nakanishi K. (2015). High-level doping of nitrogen, phosphorus, and sulfur into activated carbon monoliths and their electrochemical capacitances. Chem. Mater..

[56] Fedoseeva Y., Lapteva L.L., Makarova A.A., Bulusheva L.G., Okotrub A.V. (2018). Charge polarization in partially lithiated single-walled carbon nanotubes. Phys. Chem. Chem. Phys..

[57] Zatsepin D.A., MacK P., Wright A.E., Schmidt B., Fitting H.J. (2011). XPS analysis and valence band structure of a low-dimensional SiO_2_/Si system after Si^+^ ion implantation. Phys. Status Solidi Appl. Mater. Sci..

[58] Bulusheva L.G., Okotrub A.V., Kuznetsov V.L., Vyalikh D.V. (2007). Soft X-ray spectroscopy and quantum chemistry characterization of defects in onion-like carbon produced by nanodiamond annealing. Diam. Relat. Mater..

[59] Fedoseeva Y.V., Okotrub A.V., Koroteev V.O., Borzdov Y.M., Palyanov Y.N., Shubin Y.V., Maksimovskiy E.A., Makarova A.A., Münchgesang W., Bulusheva L.G. (2019). Graphitization of ^13^C enriched fine-grained graphitic material under high-pressure annealing. Carbon N. Y..

[60] Pacilé D., Meyer J.C., Fraile Rodríguez A., Papagno M., Gómez-Navarro C., Sundaram R.S., Burghard M., Kern K., Carbone C., Kaiser U. (2011). Electronic properties and atomic structure of graphene oxide membranes. Carbon N. Y..

[61] Iyer G.R.S., Wang J., Wells G., Bradley M.P., Borondics F. (2015). Nanoscale imaging of freestanding nitrogen doped single layer graphene. Nanoscale.

[62] Lapteva L.L., Fedoseeva Y.V., Shlyakhova E.V., Makarova A.A., Bulusheva L.G., Okotrub A.V. (2019). NEXAFS spectroscopy study of lithium interaction with nitrogen incorporated in porous graphitic material. J. Mater. Sci..

[63] Hua W., Gao B., Li S., Ågren H., Luo Y. (2010). X-ray absorption spectra of graphene from first-principles simulations. Phys. Rev. B..

[64] Piazza A., Giannazzo F., Buscarino G., Fisichella G., La Magna A., Roccaforte F., Cannas M., Gelardi F.M., Agnello S. (2016). Effect of air on oxygen p-doped graphene on SiO_2_. Phys. Status Solidi Appl. Mater. Sci..

[65] Fedoseeva Y.V., Gorodetskiy D.V., Baskakova K.I., Asanov I.P., Bulusheva L.G., Makarova A.A., Yudin I.B., Plotnikov M.Y., Emelyanov A.A., Rebrov A.K. (2020). Structure of diamond films grown using high-speed flow of a thermally activated CH_4_-H_2_ gas mixture. Materials.

[66] Matsoso B.J., Ranganathan K., Mutuma B.K., Lerotholi T., Jones G., Coville N.J. (2016). Time-dependent evolution of the nitrogen configurations in N-doped graphene films. RSC Adv..

[67] Kim K.J., Yang S., Park Y., Lee M., Kim B., Lee H. (2013). Annealing effects after nitrogen ion casting on monolayer and multilayer graphene. J. Phys. Chem. C.

[68] Usachov D., Fedorov A., Vilkov O., Senkovskiy B., Adamchuk V.K., Yashina L.V., Volykhov A.A., Farjam M., Verbitskiy N.I., Grüneis A. (2014). The chemistry of imperfections in N-graphene. Nano Lett..

[69] Isacsson A., Cummings A.W., Colombo L., Colombo L., Kinaret J.M., Roche S. (2017). Scaling properties of polycrystalline graphene: A review. 2D Mater..

[70] Duan J., Chen S., Jaroniec M., Qiao S.Z. (2015). Heteroatom-doped graphene-based materials for energy-relevant electrocatalytic processes. ACS Catal..

[71] Pham T.V., Kim J.-G., Jung J.Y., Kim J.H., Cho H., Seo T.H., Lee H., Kim N.D., Kim M.J. (2019). High areal capacitance of N-doped graphene synthesized by arc discharge. Adv. Funct. Mater..

[72] Ito Y., Christodoulou C., Nardi M.V., Koch N., Sachdev H., Müllen K. (2014). Chemical vapor deposition of N-doped graphene and carbon films: The role of precursors ans gas phase. ACS Nano.

[73] Lu Y.-F., Lo S.-T., Lin J.-C., Zhang W., Lu J.-Y., Liu F.-H., Tseng C.-M., Lee Y.-H., Liang C.-T., Li L.-J. (2013). Nitrogen-doped graphene sheets grown by chemical vapor deposition: Synthesis and influence of nitrogen impurities on carrier transport. ACS Nano.

[74] Sedelnikova O.V., Fedoseeva Yu.V., Romanenko A.I., Dusel’nikov A.V., Vilkov O.Yu., Maksimovskiy E.A., Bychanok D.S., Kuzhir P.P., Bulusheva L.G., Okotrub A.V. (2019). Effect of boron and nitrogen additives on structure and transport properties of arc-produced carbon. Carbon.

